# Improving psychiatric nurses’ competencies in spiritual care and integration of clients’ religion/spirituality into mental healthcare: outcomes of an online spiritual care training program

**DOI:** 10.1186/s12888-022-04280-9

**Published:** 2022-10-14

**Authors:** Mahbobeh Shamsi, Zohreh Khoshnood, Jamileh Farokhzadian

**Affiliations:** 1 Student Research Committee, Razi Faculty of Nursing and Midwifery, Kerman University of Medical Sciences, Kerman, Iran; 2 Department of Community Health Nursing, Razi Faculty of Nursing and Midwifery, Kerman University of Medical Sciences, Kerman, Iran; 3 Nursing research center, Kerman University of medical science, Kerman, Iran

**Keywords:** Spiritual care, Education, Religion/spirituality, Mental health nursing, Spiritual care competence

## Abstract

**Background:**

Religion/spirituality (R/S), which is associated with individuals’ well-being and psychological health, plays a significant role in most clients’ lives in healthcare systems. Although clients in mental healthcare settings prefer their R/S to be employed in nursing care, R/S has neither been adequately integrated into mental healthcare nor discussed in the assessment and nursing interventions of mental healthcare. Evidence shows that most psychiatric nurses receive little or no training in spiritual care (SC) and are unable to integrate clients’ R/S into mental healthcare. To address this gap, the present study aimed to investigate the effects of an online SC training program on psychiatric nurses’ competencies in SC and the integration of clients’ R/S into mental healthcare.

**Methods:**

This experimental study was conducted with nurses working in a psychiatric hospital affiliated with a large University of Medical Sciences in southeast Iran. Random sampling was performed and 95 nurses were assigned to the intervention (n = 50) and control (n = 45) groups. Online SC training was conducted for the intervention group in four sessions over four weeks. Data were collected using the Self-Assessment of Spiritual Care Competency and R/S Integrated Practice Assessment Scale before and one month after the training program.

**Results:**

There were no significant differences between the two groups before training (p > 0.05). After the training, nurses in the intervention group obtained significantly higher scores in competencies in SC and integration of clients’ R/S into mental healthcare compared to the control group, with a considerable effect size (P < 0.05).

**Conclusion:**

The online training program positively affected psychiatric nurses’ competencies in SC and the integration of clients’ R/S into mental healthcare. Since SC is a critical need for clients, specifically in mental healthcare settings, nurses must receive continuous education to provide SC to various clients.

## Introduction

Recently, religion/spirituality (R/S), a significant component of clients’ lives, has been investigated at an exponential rate [1, 2]. Shreds of evidence indicate that the integration of clients’ R/S in mental healthcare may improve their well-being and mental health outcomes [3–5]. As a result, many healthcare professionals, such as psychiatric nurses, have focused on the potential integration of clients’ R/S in mental healthcare [1, 2]. In other words, psychiatric nurses should be prepared to ethically address R/S issues and spiritual care (SC) as part of holistic and patient-centered care in mental health settings and to gain the required competencies in SC and integration of clients’ R/S into practice [6].

The concept of SC extends beyond psychosocial care. In other words, it facilitates maintaining all existential dimensions of clients’ lives by conducting meaning, presence, support, or care-oriented interventions. This helps clients find meaning and purpose in a disease because of inner peace. In response to spiritual needs, the SC is considered to recognize what clients have not received. Spirituality and its nature in nursing are associated with the concept of competencies in SC and the integration of clients’ S/R into practice, which are defined as the knowledge, skills, and attitudes required to develop SC [7]. Another definition maintains that competencies in SC and integration of clients’ S/R into practice include nursing interventions or activities that help clients achieve physical, mental, social, and spiritual well-being [8]. Instances of activities and interventions include determining and meeting clients’ R/S needs by respecting their personal differences, facilitating the individuals’ participation in religious gatherings, developing effective communication by careful listening to and talking with clients, taking care of clients and supporting them compassionately by showing empathy, creating a sense of well-being in patients by helping them to find meaning and purpose in their life, and referring them to other R/S care professionals such as chaplains to receive specialized SC [9, 10].

In collaboration with the clients’ family members and R/S care providers, such as chaplains, psychiatric nurses and healthcare professionals play a significant role in meeting the clients’ R/S needs. The impaired holistic care has been criticized because the spiritual dimension has often been overlooked by healthcare professionals. This can be explained by feelings of incompetence caused by lack of education in the fileds of SC, inter-professional training, work overload, time limitations, variety of cultures, personal spirituality, ethical issues, and willingness to deliver SC [10, 11]. Unqualified psychiatric nurses may engage in unethical practices that undermine their role in clients’ spiritual belief systems and impair the clients’ coping skills when faced with problems. Therefore, psychiatric nurses are required to respect the beliefs and cultural customs of clients and try to meet their R/S needs, even if they are contrary to their own beliefs [12]. Clients also prefer nurses in mental healthcare settings to be aware of their R/S beliefs, involve them in nursing planning and interventions, and talk about the impact of R/S beliefs on the healing process [13].

A literature review noted that most nurses are aware of the significance of providing SC and show positive attitudes towards SC and integration of clients’ R/S into mental healthcare. They recognized SC as one of the principles of evidence-based care, cultural care, ethical codes of nursing, and accreditation standards in the field of education [13, 14]. However, SC and the integration of clients’ R/S into nursing practice have not been studied adequately because nurses have not received the necessary education and are not adequately qualified in this field. Therefore, empowerment programs for nurses should be prioritized to enhance SC provision and clients’ spiritual comfort [1, 15, 16]. According to the literature in Iran, attitudes towards SC and SC competencies among nurses are not at an appropriate level. Training programs usually have a short-term effect caused by individuals’ forgetfulness, especially in nursing occupations that are heavily loaded. In this vein, the integration of a spiritual content-based curriculum and clients’ R/S into nursing academic programs and in-service training is essential [11, 17–21].

The limited number of studies on the effectiveness of training programs on nurses worldwide indicated enhancement in competencies of nurses in SC and integration of clients’ R/S in clinical practice in the intervention group [9, 22, 23]. To the best of our knowledge, no study has investigated the effectiveness of SC training on psychiatric nurses’ competencies. Only one study in the U.S.A concluded that an online training program had a positive impact on competencies in SC and integration of clients’ R/S into practice among mental health professionals (in the areas of psychology, psychiatry, marriage and family therapy, clinical social work, and professional counseling). Researchers have emphasized that R/S is a part of multicultural care, but has not yet been fully addressed in mental health education programs. When ignoring this type of training, many mental healthcare providers lack the competency and self-efficacy needed for SC [1]. Moreover, other studies on mental health clients’ attitudes towards SC and integrating R/S into treatment emphasized the necessity of training programs to improve mental healthcare providers’ competencies in this field [1, 6, 16].

Given R/S as a critical aspect of multicultural diversity in mental health clients, the related literature faces a paucity of studies on designing and evaluating SC training programs to empower psychiatric nurses in SC and integration clients’ R/S into practice in multi-cultural contexts [1, 13, 15]. Despite these professional requirements, psychiatric nurses do not receive appropriate training in the field of SC or integration of R/S approaches into mental healthcare. Consequently, the present study aimed to investigate the effects of an online SC training program on psychiatric nurses’ competencies in SC and the integration of clients’ R/S into mental healthcare.

## Methods

### Study design and settings

The present experimental study was conducted using a pretest-posttest design with intervention and control groups. The participants were selected from a psychiatric hospital affiliated with Kerman University of Medical Sciences in southeast Iran.

### Target population and sampling

All nurses (N = 120) working in the above-mentioned hospital at the time of data collection were considered the target population using the census method. Based on the inclusion criteria, nurses who were willing to participate in the study and had a bachelor’s or master’s degree with at least six months of work experience in the psychiatric hospital entered the study. The exclusion criteria included not attending the SC training for more than one session, taking a leave or transferring to another hospital, and being exposed to major stressors, such as divorce and death of a family member or friend. To conduct the study, the first researcher obtained the list of all nurses (with their phone numbers) working in the psychiatric ward from the nursing management office and contacted them to participate in the study. According to the inclusion criteria, 100 nurses volunteered to participate in the study and were allocated to the intervention (n = 50) and control (n = 50) groups using a table of random numbers. Finally, 95 nurses completed the questionnaires; five nurses from the control group were excluded because they did not complete the questionnaires in the post-intervention administration (response rate = 95%).

### Assessment instruments

Participants’ demographic and R/S information were collected using a 13-item form. In addition, nurses’ SC competencies were evaluated using the standardized self-assessment SC Competency Scale (SCCS) and R/S Integrated Practice Assessment Scale (RSIPAS).

**Demographic and R/S information form**: Psychiatric nurses reported their age group, gender, marital status, work position, type of employment, shift work, type of ward, education degree, work experience (years), attendance at previous training on SC in mental healthcare, degree of religiosity, degree of spirituality, and frequency of providing SC for patients (see Table 1).

**Table 1:** Demographic and R/S information of the two study groups

Variables	Categories	Intervention		Control		*X* ^2^	*P*-value
		n	%	n	%		
Age groups	< 30	11	22	14	31.1	2.10	0.35
	30–40	19	38	19	42.2		
	> 40	20	40	12	26.7		
Gender	Male	8	16	12	26.7	1.62	0.20
	Female	42	84	33	73.3		
Marital status	Single	5	10	12	26.7	5.20	0.07
	Married	44	88	33	73.3		
	Other	1	2	0	0		
Work position	Nurse	40	80	36	80	0.53	0.91
	Head nurse	5	10	4	8.9		
	Supervisors	4	8	3	6.7		
	Other	1	2	2	4.4		
Type of employment	Committed	8	16	7	15.6	5. 90	0.11
	Temporary-to-permanent	6	12	0	0		
	contract recruiters	10	20	10	22.2		
	Hired (permanent)	26	52	28	62.2		
Shift work	Fix	6	12	9	20	7.03	0.12
	Rotation	44	88	36	80		
Type of ward	Emergency and children	12	24	7	15.5	7.03	0.13
	Women	12	24	13	28.9		
	Men	22	44	21	46.7		
	Supervisory	4	8	3	6.7		
	Electroconvulsive therapy (ECT)	0	0	1	2.2		
Education degree	Bachelor	41	82	38	84.4	0.10	0.75
	Master	9	18	7	15.6		
Work experience (years)	˂10	15	30	17	37.8	0.75	0.68
	10–20	31	62	24	53.3		
	> 20	4	8	4	8.9		
Attendance at previous training on SC	Yes	5	10	4	8.9	0.034	0.85
	No	45	90	41	91.1		
Degree of religiosity	High	7	14	8	17.8	2.05	0.56
	Moderately	31	62	31	68.9		
	Slightly	11	22	5	11.1		
	Not at all	1	2	1	2.2		
Degree of spirituality	high	6	12	8	17.8	2.91	0.23
	Moderately	32	64	32	71.1		
	Slightly	12	24	5	11.1		
	Not at all	0	0	0	0		
Providing SC for patients	low	5	10	1	2.2	3.14	0.53
	Moderate	26	52	24	53.3		
	High	16	32	18	40		
	Very high	3	6	2	4.4		

**SCCS**: This scale was administered to assess the effects of the training program on nurses’ SC competencies. The SCCS includes six subscales and 27 items: (a) assessment and implementation of SC (6 items), (b) professionalization and improving quality of care (6 items), (c) personal support and counseling of patients (6 items), (d) referral to other professionals (3 items), (e) attitudes toward patients’ spirituality (4 items), and (f) communication (2 items). The nurses were required to answer the SCCS items on a 5point Likert scale, with options ranging from strongly disagree, disagree, neutral, agree, and strongly agree. Possible attainable scores ranged from 27 to 135, with higher scores indicating higher levels of competencies in the SC. The content validity of the SCCS was confirmed based on opinions provided by a panel of experts. Reliability of SCCS was also corroborated using internal consistency and Cronbach’s alpha of all subscales: assessment and implementation of SC (α = 0.82), professionalization and improving quality of care (α = 0.82), personal support and counseling of patients (α = 0.81), referral to other professionals (α = 0.79), attitudes toward patients’ spirituality (α = 0.56), and communication (α = 0.71) subscales [24]. The Persian version of the SCCS received content validity through factor analysis and its reliability was authenticated by internal consistency via the overall Cronbach’s alpha of 0.77. Internal consistency of the subscales was also supported: assessment and implementation of SC (α = 0.85), professionalization and improving quality of care (α = 0. 82), personal support and counseling of patients (α = 0. 76), referral to other professionals (α = 0.83), attitudes toward patients’ spirituality (α = 0.72), and communication (α = 0.65) [25].

**RSIPAS**: The RSIPAS was developed to measure practitioners’ competency and overall orientation in integrating clients’ R/S into practice [15, 26]. It includes four subscales and 40 items: (a) self-efficacy in integrating clients’ R/S into practice (13 items), (b) attitudes towards integrating clients’ R/S into practice (12 items), (c) perceived feasibility of engaging in R/S integrated practice (six items), and (d) behaviors associated with integrating clients’ R/S into practice (nine items). In the first three subscales, the respondents were required to answer the items on a 5point Likert scale using the options of “strongly disagree, disagree, neutral, agree, and strongly agree”. The subscale related to respondents’ behaviors ranged from never, rarely, sometimes, often, to very often. Item 12 in the attitude subscale, as well as items 3 and 4 in the feasibility subscale, had negatively worded stems and were reverse-scored in inferential analyses. The lowest and highest attainable scores were 40 and 200, respectively; higher scores indicated higher levels of competency in integrating clients’ R/S into practice. The content validity of the RSIPAS was confirmed based on opinions obtained from a panel of experts. The validity of the scale (criterion, discriminant, construct, and factor analysis) was investigated and corroborated. Moreover, the reliability of the RSIPAS was confirmed, with an overall Cronbach’s alpha of 0.95. Internal consistency of self-efficacy (α = 0.91), attitudes (α = 0.88), behaviors (α = 0.87) perceived feasibility (α = 0.88) subscales were also corroborated [15].

The Persian version of RSIPAS received content validity confirmation from 10 nursing faculty members and its reliability was supported for the whole scale (overall internal consistency Cronbach’s alpha = 0.91) and its subscales: internal consistency of self-efficacy (α = 0.89), attitudes (α = 0. 81), behaviors (α = 0.76) perceived feasibility (α = 0.91) subscales were corroborated [21].

### Data collection and evaluation period

Data collection and training program were conducted between September and November 2021. Two weeks prior to the implementation of the training program, separate WhatsApp channels were created for the intervention and control groups by the first researcher. Nurses enrolled in the study were asked to study the purpose and process of the training program and sign an informed consent form. In the baseline assessment (pre-test), the questionnaire link was sent to the WhatsApp group, and the participants were instructed to complete the questionnaires. Later, nurses in the intervention group were provided with explanations about the details of the training program and its duration. After setting an appropriate time for the educational sessions, the first researcher ensured that the nurses received a valid certificate after completing the training program. To attain the highest participation rate in the intervention group, the training group was divided into two groups of 25 members. They were offered two alternative times for each training session, and they could choose the appropriate day based on their work shifts and schedules. Furthermore, if nurses could not attend a session, they could attend the alternative session within the same week.

One month after implementing the SC training (post-test), the SCCS and RSIPAS were readministered to all participants, and their results were compared to those achieved in the pretest to evaluate the efficiency of the SC training curriculum.

### Designing and implementing the SC training program

To develop the curriculum of SC training program, core competencies in SC and integration of clients’ R/S into the nurses’ practices, especially those working in the area of mental healthcare were considered [1, 2, 10, 14, 21–23, 27–31]. Then researchers consulted with a qualified multidisciplinary team in SC consisting of a psychiatrist, a psychologist, a psychiatric nurse, a P.h.D in nursing, a counselor at clinical mental health, and a Muslim chaplain working at the Spiritual Health Center affiliated with Kerman University of Medical Sciences. The focus group method was employed to conduct this consultation. The team members were asked to present their experiences and viewpoints on the required training contents and core competencies of the clinical and psychiatric nurses concerning SC. Furthermore, the instruments applied in the present study to assess the core competencies were examined. At this stage, the topics were developed using focus group discussions and reviewed for suitability, applicability, and relevance to the nursing process (assessment, nursing diagnosis, interventions, and evaluation of SC) by the research team. Followed by reaching an agreement on the core competencies, the researcher drafted the program contents. In order to investigate content validity of the educational content, an educational supervisor, two nurses from mental health fields, as well as four nursing faculty members, who were blind to the research purpose were asked to provide their feedbacks on the course content.

The proposed training curriculum was implemented by the project manager (JF) according to her academic certificate, experiences in teaching, and publications in the area of SC. The program was taught in four two-hour sessions for four weeks via an online method (synchronously/lives) using a sky room (web video conferencing platform). The participants were also provided with educational materials offline, including recorded video and audio files of each session via the WhatsApp channel. The nurses were able to review the training materials and discuss their probable questions with the research team through WhatsApp. Some assignments were also administered to the participants to complete and upload the WhatsApp groups. The researchers provided nurses with feedback on their assignments using WhatsApp.

Reminders were sent to encourage the participants to study the already covered materials, complete the assigned homework, and attend the next session. To prevent treatment diffusion between the intervention and control groups, the intervention group members were asked not to share educational content with the control group until the end of the study. The themes covered in SC training curriculum are represented in Table 2.

**Table 2:** Themes covered in the SC training curriculum

Goal: To improve the psychiatric nurses’ competencies in SC and integration of clients’ R/S into clinical practice.		
**Target group**: Psychiatric nurses in the intervention group (Group 1 = 25 and Group 2 = 25) **Expected duration**: Four two-hour sessions in four weeks **Training methods**: Lectures and tutorials, PowerPoint presentations, case reports sharing, Video clips and short movies, audio-visual materials, group discussion, role playing, Web-based learning, oral reports and storytelling, contributions of work-based experiences, questions and answers, assignments, book reading about SC **Evaluation method**: Pre-test and post-test scores obtained from SCCS and RSIPAS were compared.		
**Sessions**	**Main topic**	**Content**
1	Introduction and attitudes towards SC and integration clients’ R/S into clinical practice	- Introduction and explanation of the goals and expectations related to the SC training - Definition of R/S concepts; similarities and differences - Spiritual health and R/S needs of clients in mental healthcare, and R/S diversity in clients -Effects of the spiritual health on other dimensions of health - The importance of spiritually and SC in mental healthcare - Definition and outcomes of SC and integration of client’s R/S in to mental healthcare - History and condition of SC in mental healthcare and national policies on providing SC care - Definition of concepts like forgiveness, thankfulness, mindfulness, presence, hope, meaning, connection to transcendent power, spiritual change, and ultimate reality. -Orientation toward the role of chaplains and R/S leaders, as members of the multidisciplinary team in providing SC - Barriers and challenges of SC and integration of client’s R/S in mental healthcare settings - Definition of competencies for SC and integration of clients’ R/S into clinical practice - Assignment: Writing examples and case reports based on the psychiatric nurses’ clinical experiences to assess R/S in clients in mental healthcare settings (as the first step in nursing process to discuss about it in next sessions)
2	Integration of SC in the nursing process (Assessing spirituality in mental healthcare)	- Assessment of spiritual needs of clients in mental healthcare settings -Self-consciousness and self-assessment, identification of limitations of the nurses’ skills with regard to assessing clients’ mental healthcare - Identification of the methods for assessing clients’ R/S and collection of the clients’ spiritual history (standardized assessment tools, manualized interventions, empirically supported practical behaviors related to clients’ characteristics and preferences) - Assessment of the clients’ reactions to the feelings of loneliness, weakness, disease, and death -Introduction of some question examples to assess and take history from the clients’ spiritual and religious beliefs/values - Recognition of the clients’ R/S sources and mental healthcare settings -Distinguishing between spiritual experiences and psychopathology - Assignment: Discuss about the case reports and experiences of nurses, R/S assessment in the cases suggested by nurses, administration of the questionnaires and model suggested by instructors.
3	Integration of SC in the nursing process (nursing diagnosis and problems and interventions)	- Nursing diagnosis/problems and implementation of SC interventions: - Recognition of the nursing diagnosis and R/S problems of the clients in the diagnostic and statistical manual of mental disorders - Use of verbal/non-verbal skills related to the clients’ culture - Use of communication skills with respect to SC - Recognition of positive and negative R/S coping strategies - Spiritual distress indicators and verbal statements of the clients with regard to spiritual distress - Planning for the implementation of R/S care and applying the principles of R/S services - Nurses’ application of the necessary skills to provide R/S interventions, such as active and compassionate communication, humor, touch, music therapy, and self-awareness - Interventions to reduce spiritual distress, decrease R/S problems, and promote spiritual growth of the clients by cooperation other R/S care professionals, such as chaplains/pastors - Recognition of R/S conflicts, personal needs of patients in relation to R/S - Reffering the clients/their families to members of the multidisciplinary team (e.g., chaplains and R/S leaders) timely in the case of requiring help in their theological beliefs and rise of conflicts -Having awarrness about personal limitations in providing SC and consulting with other members of the multidisciplinary team (e.g., psychologists, chaplains, R/S leaders, and counsellors) as deemed necessary - Introducing R/S resources, supporting systems/agencies to clients and their families (e.g., place for worship and support groups) - Application of the clinical guidelines localized for SC and clients’ R/S in to nursing in Iran - Assignment: determining problems in spiritual diagnoses in case reports as well as designing appropriate interventions for such problems
4	Integration of SC in the nursing process (Evaluation and documentation)	- Evaluation of the SC outcomes for the clients in mental healthcare - Application of the evaluation methods for the R/S interventions - Evaluation of variables related to clients, nurses, and treatment process that affect the appropriateness and effectiveness of spiritual interventions in mental healthcare - Documentation of the SC integration in the nursing process and composition of comprehensive nursing notes based on the holistic care - Limitations / barriers and ethical challenges in the SC at mental healthcare settings - Reflection on the personal experiences related to the aspects of SC and integration of the clients’ R/S in mental healthcare settings - Planning for clients’ discharge and follow-up on their R/S problems in home care - Ensuring about the follow-up by providing SC feedback to clients and other stakeholders - Summary and conclusion of the training program - Assignment: A review of the case reports designed in previous sessions, nursing process, and evaluation of the SC.

### Statistical analysis

Data analysis was performed using SPSS version 21 (frequency, percentage, mean score, and standard deviation) and inferential statistics (chi-square, independent t-test, paired t-test, and analysis of covariance, and multivariate linear regression). The Kolmogorov-Smirnov test showed a normal data distribution. The significance level was set at P ≤ 0.05.

## Results

### Participants’ characteristics

Table 1 shows the participants’ demographic and R/S information for the intervention and control groups. A total of 95 nurses completed the study and returned the questionnaires. Most participants in the intervention and control groups were nurses (88, 88%) within the age range of 30–40 years (38, 42.2%), female (84, 73.3%), married (88, 73.3%), worked in men’s ward (44, 46.7%), had permanent employment (52, 62.2%), rotational working shifts (88, 80%), and bachelor’s degree (80, 84.4%) with a working experience of 10–20 years (62, 53.3%). The majority of nurses were at moderate levels of being religious (62, 68.9%) and spiritual (64, 71.1%), did not attend previous training on SC in mental healthcare (90, 91.1%), and provided SC to patients at moderate levels (52, 53.3%). The two study groups were homogenous in terms of demographics and R/S information.

### Comparison of changes in competencies based on SCCS

Based on the between-group comparisons calculated using independent t- test, the intervention (70.62 ± 17.52) and control (65.30 ± 12.67) groups were not significantly different before the training in terms of their total SCCS scores (t = 1.63, *p* = 0.11). After the training, the SCCS total scores increased significantly in the intervention (125.32 ± 12.87) group compared with the control (63.59 ± 21.7) with a very large effect value (Cohen’s d = 3.46, t = 16.89, *p* = 0.001). Moreover, the intervention group had higher scores on all subscales of the SCCS than did the control group; the effect sizes attributed to the subscale scores ranged from 1.38 to 3.32 in the intervention group after the training.

According to the within-group comparisons calculated using paired t-test, the total scores of the SCCS and its subscales improved in the intervention group after training compared with their scores prior to training. The mean differences indicated that the training program had the greatest impact on the professionalization and improving quality of care subscale (M diff = 30.84), whereas the lowest impact was attributed to the communication subscale (M diff = 3.38) (Table 3).

**Table 3:** Comparison of competency scores in the intervention and control groups for SC based on SCCS before and after the SC training

Variable	Time	Prior to the intervention	After the intervention	Within group differences	ES^*^ (Cohen’s d)	Paired *t*-test	*P*- value
	**Groups**	**M ± SD**	**M ± SD**				
Assessment and implementation of SC	Intervention	14.28 ± 3.98	27.3 ± 3.02	13.02	3.68	18.09	0.001*
	Control	13.09 ± 3.24	13.5 ± 5.34	0.78	0.15	-0.93	0.36
	Independent *t*- test	1.54	14.78				
	*P*-value	0.126	0.001*				
	ES^*^ (Cohen’s d)	0.32	1.38				
Professionalization and improving quality of care	Intervention	14.6 ± 4.31	27.82 ± 3.20	13.22	3.48	17.92	0.001*
	Control	13.30 ± 3.65	13.5 ± 5.34	0.19	0.04	-0.23	0.81
	Independent *t*- test	1.53	15.85				
	*P*-value	0.13	0.001*				
	ES^*^ (Cohen’s d)	0.32	3.25				
Personal support and counseling of patients	Intervention	15.76 ± 4.78	27.98 ± 3.22	12.22	2.99	15.48	**0.001** ^*^
	Control	15.2 ± 3.43	13.83 ± 5.08	1.19	0.27	1.51	0.14
	Independent *t*- test	0.83	16.17				
	*P*-value	0.407	**0.001** ^*^				
	ES^*^ (Cohen’s d)	0.17	3.32				
Referral to other professionals	Intervention	8 ± 2.42	13.96 ± 1.76	5.96	2.81	15.77	**0.001** ^*^
	Control	7.42 ± 1.95	6.95 ± 2.51	0.47	0.20	1.57	0.124
	Independent *t*- test	1.22	15.65				
	*P*-value	0.22	**0.001** ^*^				
	ES^*^ (Cohen’s d)	0.26	3.23				
Attitudes toward patients’ spirituality	Intervention	13.7 ± 1.69	18.8 ± 1.88	5.10	2.84	3.12	**0.003** ^*^
	Control	10.9 ± 2.81	10.14 ± 3.43	0.76	0.24	1.1	0.275
	Independent *t*- test	1.51	15.30				
	*P*-value	0.134	**0.001** ^*^				
	ES^*^ (Cohen’s d)	0.32	3.13				
Communication	Intervention	6.08 ± 1.73	9.46 ± 3.13	3.38	1.25	11.95	**0.001** ^*^
	Control	5.54 ± 1.21	5.28 ± 1.61	0.26	0.18	0.93	0.35
	Independent *t*- test	1.67	16.04				
	*P*-value	0.09	**0.001** ^*^				
	ES^*^ (Cohen’s d)	0.09	1.67				
Total of the SCCS	Intervention	70.62 ± 17.52	125.32 ± 12.87	54.7	3.55	19.43	**0.001** ^*^
	Control	65.30 ± 12.67	63.59 ± 21.7	4.33	0.09	1.71	0.59
	Independent *t*- test	1.63	16.89				
	*P*-value	0.11	**0.001** ^*^				
	ES^*^ (Cohen’s d)	0.34	3.46				

*Bold p-values are significant at the level of ≤ 0.05, Effect size (ES):0-0.2 = small effect, 0.2–0.5 = moderate effect, > 0.5–0.7 = large effect; and > 0.7 = very large effect

### Comparison of changes of competencies based on RSIPAS

Between-group comparisons before training, calculated using independent t- test, indicated that the total RSIPAS scores were not significantly different between the intervention (99.74 ± 20.90) and control (94.97 ± 17.57) groups (t = 1.17, *p* = 0.24). After the training, the RSIPAS scores were significantly higher in the intervention (169.06 ± 14.10) than in the control (95.97 ± 28.15) with very large effect values (Cohen’s d = 2.28, t = 16.11, *p* = 0.001). The results also indicated that the intervention group had higher scores in subscales of RSIPAS compared with the control group; the effect sizes related to the RSIPAS sub-scales in the intervention group ranged from 0.59 to 7.67 after training.

Based on within-group comparisons calculated using paired t-test, the intervention group’s RSIPAS scores and its subcategories, including self-efficacy, attitudes, and behaviors, increased significantly after the training compared with the before the intervention scores. Mean differences suggest that the training program had the highest effect on the self-efficacy subscale (M diff *=* 13.22) and its lowest impact on participants’ attitudes (M diff *=* 17.74). However, the perceived feasibility subscale did not increase significantly after the training compared to that before the intervention (*p* = 0.19) (Table 4).

**Table 4:** Comparison of competency scores of the intervention and control groups based on RSIPAS before and after the SC training

Variable	Time	Prior to the intervention	After the intervention	Within group differences	ES^*^ (Cohen’s d)	Paired t-test	*P*- value
	**Groups**	**M ± SD**	**M ± SD**				
Self-efficacy	Intervention	11.55 **±** 27.98	6.33 **±** 58.82	30.84	3.31	-16.60	0.001*
	Control	7.63 **±** 26.71	11.59 **±** 29.76	3.04	0.31	-1.67	0.10
	Independent t-test	0.607	15.23				
	*P*-value	0.54	**0.001**				
	ES^*^ (Cohen’s d)	0.12	3.11				
Attitudes	Intervention	10.64 **±** 34.46	4.59 **±** 52.20	17.74	2.16	-11.8	0.22
	Control	9.90 **±** 30.66	9.61 **±** 28.78	1.88	0.19	1.07	0.28
	Independent t- test	1.75	15.27				
	*P*-value	0.08	**0.001** ^*^				
	ES^*^ (Cohen’s d)	0.36	3.10				
Behaviors	Intervention	8.12 **±** 20.76	4.10 **±** 40.26	19.5	3.03	-15.11	**0.001** ^*^
	Control	5.52 **±** 18.61	1.14 **±** 17.16	1.64	0.36	-1.21	0.23
	Independent t- test	1.44	2.83				
	*P*-value	0.15	**0.006** ^*^				
	ES^*^ (Cohen’s d)	0.30	7.67				
Perceived feasibility	Intervention	6.49 ± 16.54	0.93 ± 17.78	1.24	0.26	-1.32	0.19
	Control	9.58 ± 18.97	1.14 **±** 17.16	1.80	0.26	1.18	0.24
	Independent t- test	-1.44	16.44				
	*P*-value	0.15	**0.001** ^*^				
	ES^*^ (Cohen’s d)	0.29	0.59				
Total of RSIPAS	Intervention	20.90 **±** 99.74	14.10 **±** 169.06	69.32	3.88	-19.79	0.001*
	Control	17.57 **±** 94.97	28.15 ± 95.97	1	0.04	-0.22	0.82
	Independent t- test	1.17	16.11				
	*P*-value	0.24	0.001*				
	ES^*^ (Cohen’s d)	0.24	3.28				

^*^Bold p-values are significant at the level of ≤ 0.05, Effect size (ES):0-0.2 = small effect, 0.2–0.5 = moderate effect, > 0.5–0.7 = large effect; and > 0.7 = very large effect

In addition, the covariance analysis test was used to control and check the effects of pretest on nursing students’ scores SCCS and RSIPAS. The results showed a statistically significant difference in the total posttest scores of SCCS and RSIPAS between the control and intervention groups (Table 5). These results are also consistent with the results of Tables 3 and 4.

**Table 5:** Summary of covariance analysis for the two groups of control and intervention

		Type III sum of squares	Df	Mean square	F	*P*-value
	Intercept	24724.14	2	24724.14	86.09	0.001*
SCCS	Pretest	866.33	1	866.33	0.49	0.35
	Group	80239.11	1	80239.11	279.40	0.001*
	Error	25558.66	89	287.17		
	Intercept	44262.36	2	44262.36	96.30	0.001*
RSIPAS	Pretest	1349.35	1	1349.35	2.93	0.09
	Group	116997.69	1	116997.69	254.55	0.001*
	Error	40906.44	89	459.62		

*Bold p-values are significant at the level of ≤ 0.05

### Multivariate linear regression

Following the application of multivariate linear regression, the demographic and R/S variables of psychiatric nurses could not predict the SCCS and RSIPAS score changes significantly. Consequently, the effectiveness of the training program was not dependent on the participants’ demographic and R/S variables.

## Discussion

The present study evaluated the effect of an online SC training program on psychiatric nurses’ competencies in SC and integration of clients’ R/S into mental healthcare. According to the findings, SC training improved the psychiatric nurses’ self-reported scores in competencies of nurses in SC and integration of the clients’ R/S into mental healthcare significantly with a very large effect size. This result has been confirmed in several studies [9, 23, 32–35]. A systematic review assessed the outcomes of SC training for healthcare professionals in clinical and/or academic settings. The measured outcomes indicated evidence of increased competencies in SC and professional responsibilities. The results presented improvements in providing multidisciplinary SC and integrating R/S needs into practices of nurses with regard to patients and their family members. The review categorized the main objectives of SC training as developing participants’ sensitivity towards their own spirituality, clarifying the role of spirituality in healthcare, and preparing participants for spiritual encounters. This study highlighted the importance of training programs in integrating R/S into holistic care. The majority of studies reported that SC training was perceived as a beneficial intervention for making nurses aware and conscious of the role of R/S in caring for patients. The participants enjoyed the holistic nature of the designed training course and noted that the educational content could successfully deal with real-world situations [36].

Hu et al. indicated that nurses who received a 12-month educational intervention had significantly higher scores in SC competencies and spiritual well-being than the control group members, with a moderate effect size. According to the intervention group members, the training program could provide an opportunity for them to review their spiritual needs. In turning the obtained knowledge and skills into active conscious clinical practice, the nurses’ viewpoints have improved about life and death, and their attitudes have changed towards the events around them [22]. Amiri et al. conducted an online training program and noted improved levels of nurses’ competencies in integrating clients’ R/S into practice in the intervention group. As these researchers highlighted, nurses’ higher levels of competencies in SC and integration of clients’ R/S into practice can facilitate and enhance the provision of holistic care for clients. In this vein, equipping nurses with competencies in SC and integration of clients’ R/S into practice should begin with their undergraduate academic studies and continue with in-service training programs. In this regard, nurse educators are recommended to consider appropriate strategies for implementing SC training programs throughout the healthcare curriculum using new educational approaches such as virtual (online and offline) and face-to-face teaching methods [21].

Although all the above-mentioned studies evaluated the effectiveness of SC training on SC competencies of some healthcare providers, such as nurses and nursing students, no study has investigated nurses working in mental healthcare settings or psychiatric wards. In a similar study, Pearce et al. addressed the need for SC training in mental healthcare settings. They implemented an online SC training program for mental health professionals in mental health fields and evaluated its effectiveness using the SCCS and RSIPAS. Followed by the intervention, the participants’ scores of competencies in SC and integration of clients’ R/S into the mental healthcare raised significantly. According to the qualitative results of this study, the participants reported high levels of satisfaction with the online courses and their educational contents, while maintaining a decrease in the perceived barriers of integration of R/S into practice. Most participants preferred online courses and stated that they would not have completed the program if it had been in person due to some problems and limitations, such as a lack of time and resources. The participants also suggested some measures to improve the quality of these training programs: providing more educational information, case examples, discussions with peers, advanced and up-to-date topics by revising the curriculum content, organized syllabi, and longer and richer programs, setting the platform for conducting and developing future research, establishing continuous education courses for healthcare providers, and designing specific training curricula for students and trainees in the mental healthcare fields [1].

The within-group comparisons showed that the perceived feasibility subscale scores did not increase significantly after training compared to before it for the intervention group. The perceived feasibility subscale assessed the potential barriers to perform R/S integrated practices, such as the lack of adequate training to integrate R/S, time to assess the clients’ R/S background, and resources to identify potential strengths and struggles. In confirmation of these findings, a study in Iran identified nurses’ lack of time, comfort, and SC training as the main barriers to providing SC and understanding spiritual topics. Most participants indicated willingness to attend SC training courses. However, an important factor in the quality of care in Iran is the shortage of nurses. The number of nurses is not proportionate to the number of patients and challenges; for example, nursing work overload has created a permanent lack of time for nurses to provide comprehensive and holistic care. In such cases, nurses provide essential care, such as prioritization and provision of physical care over SC [37]. According to the findings, nursing managers should provide opportunities to empower nurses to integrate clients’ R/S into practice by motivating nurses to participate in training courses, creating organizational and managerial support, providing adequate time, and reducing the workload. In addition, healthcare managers and policymakers are required to employ hospital chaplains or R/S leaders as essential members collaborating in the multidisciplinary team to offer specialized SC to clients. Given that nurses did not receive the needed theological training, R/S experts with clinical pastoral can help nurses to meet the clients’ needs, enhance their theological beliefs, reduce their conflicts, and decrease their spiritual distress.

## Limitations and strengths

The present study has several limitations and recommendations. Given that the obtained results are limited to nurses working at a psychiatric hospital, the generalizability of the findings should be performed with caution. Moreover, evaluating the effectiveness of SC training was conducted based on self-assessment tools that may not provide a real picture of psychiatric nurses’ levels of competencies in SC and integration of clients’ R/S into mental healthcare. Further studies are needed to assess the generalizability of the findings to other groups of healthcare providers through larger samples and longer follow-up periods. Future studies should evaluate training programs related to SC competencies using mixed methods.

As a strength of our study, we trained nurses working in psychiatric wards because this training program is a sample of designing an SC curriculum that contributes to the literature on SC competencies in nursing education. Our experiences in designing and implementing the online SC training program can be beneficial for developing future SC programs aimed at fostering the SC competencies of in mental healthcare. Due to the heavy workload of nurses, online SC training can be a beneficial method for involving nurses in training programs.

## Conclusion

Based on these findings, the online SC training program improved psychiatric nurses’ competencies in SC and integration of clients’ R/S into mental healthcare. As a result, SC training can help bridge the gap between the lack of professional training in R/S and the real needs of mental healthcare clients. Implementation of SC program demands a multidisciplinary team approach, consisting of doctors, nurses, social workers, psychologists, counselors, R/S leaders, and chaplains. Consequently, teamwork, team learning, as well as intra- and inter-professional training should be designed and evaluated based on SC and integration of clients’ R/S into their mental treatment plan. In addition to theoretical training, practical experience is needed to assess clients’ R/S needs and to integrate their R/S into practice. To facilitate the integration process, nurses need role models and mentors to accompany this process, since they can provide positive feedback on nurses’ SC and help them improve their SC competencies. Nurse educators are also recommended to add R/S competency training courses to multi-cultural competencies. Furthermore, researchers are recommended to evaluate the feasibility and effectiveness of various training strategies in developing mental health professionals’ competencies in SC and the integration of clients’ R/S into practice in different environments and cultures. Future studies are needed to identify SC needs, challenges, barriers, facilitators, and strategies for providing appropriate SC to clients of different ethnicities, religions, and mental healthcare settings.

## Data Availability

The data are available upon request to the corresponding author after signing appropriate documents in line with ethical applications and the decision of the ethics committee.

## Abbreviations

R/S: Religion/spirituality
SC: Spiritual care
SCCS: Spiritual care competency scale
RSIPAS: R/S Integrated Practice Assessment Scale

## References

[1] Pearce MJ, Pargament KI, Oxhandler HK, Vieten C, Wong S (2020). Novel online training program improves spiritual competencies in mental health care. Spiritual Clin Pract.

[2] Oxhandler HK, Parrish DE (2018). Integrating clients’ religion/spirituality in clinical practice: A comparison among social workers, psychologists, counselors, marriage and family therapists, and nurses. J Clin Psychol.

[3] Chen J, Lin Y, Yan J, Wu Y, Hu R (2018). The effects of spiritual care on quality of life and spiritual well-being among patients with terminal illness: a systematic review. Palliat Med.

[4] Tajbakhsh F, Hosseini M, Fallahi-Khoshknab M, Rokofian A, Rahgozar M, Mary Davidson P (2018). The effect of spiritual care on depression in patients following coronary artery bypass surgery: a randomized controlled trial. Religions.

[5] Borji M, Tarjoman A (2020). Investigating the effect of religious intervention on mental vitality and sense of loneliness among the elderly referring to community healthcare centers. J Relig Health.

[6] Oxhandler HK, Pargament KI, Pearce MJ, Vieten C, Moffatt KM (2021). Current mental health clients’ attitudes regarding religion and spirituality in treatment: A national survey. Religions.

[7] Machul M, van Leeuwen R, Ozga D, Jurek K, Boczkowska S, Dobrowolska B (2022). The level of spiritual care competence of Polish nurses and the psychometric properties of the spiritual care competence scale (SCCS). BMC nurs.

[8] Cone PH, Giske T: Integrating spiritual care into nursing education and practice: strategies utilizing open journey theory. In.: *Elsevier;* 2018. 10.1016/j.nedt.2018.08.015 30216754

[9] Attard J, Baldacchino DR, Camilleri L (2014). Nurses’ and midwives’ acquisition of competency in spiritual care: A focus on education. Nurse Educ Today.

[10] Baldacchino D. Spiritual Care Education of Health Care Professionals. *Religions*.

[1] 2015, 6(594–613):129.

[11] Farahani AS, Rassouli M, Salmani N, Mojen LK, Sajjadi M, Heidarzadeh M, Masoudifar Z, Khademi F (2019). Evaluation of Health-Care Providers’ Perception of Spiritual Care and the Obstacles to Its Implementation. Asia-Pac J Oncol Nurs.

[12] Bowser D, Joseph M, Crothers LM, Kolbert JB, Holmes IS. A constructivist approach to promoting spiritual competence in counselor trainees: a pilot study. J Spiritual Ment Health 2020:1–18.

[13] Oxhandler HK, Pargament KI (2014). Social work practitioners’ integration of clients’ religion and spirituality in practice: A literature review. Soc work.

[14] Oxhandler HK (2017). Social work field instructors’ integration of religion and spirituality in clinical practice. J Soc Work Educ.

[15] Oxhandler HK, Parrish DE (2016). The development and validation of the religious/spiritually integrated practice assessment scale. Res Social Work Pract.

[16] Oxhandler HK, Ellor JW, Stanford MS (2018). Client attitudes toward integrating religion and spirituality in mental health treatment: Scale development and client responses. Soc Work.

[17] Marzband R, Hosseini SH, Hamzehgardeshi Z, Moosazadeh M (2019). Attitude of Nurses and Nursing Students to Spiritual Care in Iran: A Systematic Review and Meta-analysis. J Maz Univ Med Sci.

[18] Bar-Sela G, Schultz MJ, Elshamy K, Rassouli M, Ben-Arye E, Doumit M, Gafer N, Albashayreh A, Ghrayeb I, Turker I (2019). Training for awareness of one’s own spirituality: A key factor in overcoming barriers to the provision of spiritual care to advanced cancer patients by doctors and nurses. Palliat & supportive care.

[19] Adib-Hajbaghery M, Zehtabchi S (2014). Assessment of nurses’ professional competence in spiritual care in Kashan’s hospitals in 2014. Avicenna J Nurs Midwifery Care.

[20] Abdollahyar A, Baniasadi H, Doustmohammadi M, Sheikhbardsiri H, Yarmohammadian M (2019). Attitudes of Iranian Nurses Toward Spirituality and Spiritual Care. J Chris Nurs.

[21] Amiri H, Farokhzadian J, Tirgari B (2021). Empowerment of nurses for integrating clients’ religion/spirituality into clinical practice: outcomes of an online training program. BMC nurs.

[22] Hu Y, Jiao M, Li F (2019). Effectiveness of spiritual care training to enhance spiritual health and spiritual care competency among oncology nurses. BMC palliati care.

[23] Bulduk S, Usta E, Dinçer Y (2017). The Influence of Skill Development Training Program for Spiritual Care of Elderly Individual on Elderly Care Technician Students’ Perception of Spiritual Support. J relig health.

[24] Van Leeuwen R, Tiesinga LJ, Middel B, Post D, Jochemsen H (2009). The validity and reliability of an instrument to assess nursing competencies in spiritual care. J Clin Nurs.

[25] Khalaj M, Pakpour A, Mohammadi Zeidi I (2013). Validity and reliability of a Persian version of nursing students’ competence scale in spiritual care. J Inflamm Dis.

[26] Oxhandler HK (2019). Revalidating the religious/spiritually integrated practice assessment scale with five helping professions. Res Soc Work Pract.

[27] Osório IHS, Gonçalves LM, Pozzobon PM, Gaspar Júnior JJ, Miranda FM, Lucchetti AL, Lucchetti G (2017). Effect of an educational intervention in “spirituality and health” on knowledge, attitudes, and skills of students in health-related areas: A controlled randomized trial. Med teach.

[28] Manderino D: A constructivist approach to promoting spiritual competencies in counselor trainees. *Duquesne University, Dissertations for the degree of Doctor of Philosophy* 2014, https://dsc.duq.edu/cgi/viewcontent.cgi?article=1881&context=etd.

[29] Attard J, Ross L, Weeks KW (2019). Design and development of a spiritual care competency framework for pre-registration nurses and midwives: a modified Delphi study. Nurse Educat Pract.

[30] McSherry W, Ross L, Attard J, van Leeuwen R, Giske T, Kleiven T, Boughey A, Network E (2020). Preparing undergraduate nurses and midwives for spiritual care: Some developments in European education over the last decade. J Study Spiritual.

[31] Raffay J, Wood E, Todd A (2016). Service user views of spiritual and pastoral care (chaplaincy) in NHS mental health services: a co-produced constructivist grounded theory investigation. BMC Psychiatry.

[32] van Leeuwen R, Attard J, Ross L, Boughey A, Giske T, Kleiven T, McSherry W (2021). The development of a consensus-based spiritual care education standard for undergraduate nursing and midwifery students: An educational mixed methods study. J Adv Nurs.

[33] Momennasab M, Shadfard Z, Jaberi A, Najafi SS, Hosseini FN (2019). The Effect of Group Reflection on Nursing Students’ Spiritual Well-being and Attitude Toward Spiritual Care: a randomized controlled trial. Invest Educ Enferm.

[34] Gijsberts M-JH, Liefbroer AI, Otten R, Olsman E (2019). Spiritual care in palliative care: a systematic review of the recent European literature. Med Sci.

[35] Van de Geer J, Veeger N, Groot M, Zock H, Leget C, Prins J, Vissers K (2018). Multidisciplinary training on spiritual care for patients in palliative care trajectories improves the attitudes and competencies of hospital medical staff: Results of a quasi-experimental study. Am J Hosp Palliat Care.

[36] Paal P, Helo Y, Frick E (2015). Spiritual care training provided to healthcare professionals: a systematic review. J Pastoral Care Counsel.

[37] Farahani AS, Rassouli M, Salmani N, Mojen LK, Sajjadi M, Heidarzadeh M, Masoudifar Z, Khademi F (2019). Evaluation of health-care providers’ perception of spiritual care and the obstacles to its implementation. Asia-PacJ Oncol Nurs.

